# Cation Vacancies in Ti‐Deficient TiO_2_ Nanosheets Enable Highly Stable Trapping of Pt Single Atoms for Persistent Photocatalytic Hydrogen Evolution

**DOI:** 10.1002/smll.202502428

**Published:** 2025-06-02

**Authors:** Hayoon Jung, Gihoon Cha, Hyesung Kim, Johannes Will, Xin Zhou, Zdeněk Bad'ura, Giorgio Zoppellaro, Ana S. Dobrota, Natalia V. Skorodumova, Igor A. Pašti, Bidyut Bikash Sarma, Jochen Schmidt, Erdmann Spiecker, Josef Breu, Patrik Schmuki

**Affiliations:** ^1^ Department of Materials Science and Engineering WW4‐LKO Friedrich‐Alexander‐Universität Erlangen‐Nürnberg Martensstraße 7 91058 Erlangen Germany; ^2^ Regional Centre of Advanced Technologies and Materials Czech Advanced Technology and Research Institute (CATRIN) Palacký University Šlechtitelů 27 Olomouc 78371 Czech Republic; ^3^ Department for Correlative Microscopy and Materials Data Fraunhofer Institute for Ceramic Technologies and Systems (IKTS) Äußere Nürnberger Straße 62 91301 Forchheim Germany; ^4^ Department of Chemistry Universität Bayreuth Universitätsstraße 30 95447 Bayreuth Germany; ^5^ Institute of Micro‐ and Nanostructure Research & Center for Nanoanalysis and Electron Microscopy (CENEM) IZNF Friedrich‐Alexander‐Universität Erlangen‐Nürnberg Cauerstraße 3 91058 Erlangen Germany; ^6^ Nanotechnology Centre VŠB − Technical University of Ostrava 17. listopadu 2172/15 Ostrava‐Poruba 708 00 Czech Republic; ^7^ University of Belgrade – Faculty of Physical Chemistry Studentski trg 12–16 Belgrade 11000 Serbia; ^8^ Applied Physics Division of Materials Science Department of Engineering Sciences and Mathematics Luleå University of Technology Luleå 971 87 Sweden; ^9^ Serbian Academy of Sciences and Art Kneza Mihaila 35 Belgrade 11000 Serbia; ^10^ Laboratoire de Chimie de Coordination (LCC) CNRS Université de Toulouse INPT, UPR 8241, 205 route de Narbonne Toulouse Cedex 4 31077 France; ^11^ Institute of Particle Technology Friedrich‐Alexander‐Universität Erlangen‐Nürnberg Cauerstraße 4 91058 Erlangen Germany

**Keywords:** cation vacancy, photocatalytic hydrogen evolution, Pt single atom, TiO_2_ nanosheet

## Abstract

The stabilization of single‐atom catalysts on semiconductor substrates is pivotal for advancing photocatalysis. TiO_2_, a widely employed photocatalyst, typically stabilizes single atoms at oxygen vacancies—sites that are accessible but prone to agglomeration under illumination. Here, we demonstrate that cation vacancies in Ti‐deficient TiO_2_ nanosheets provide highly stable anchoring sites for Pt single atoms, enabling persistent photocatalytic hydrogen evolution. Ultrathin TiO_2_ nanosheets with intrinsic Ti^4+^ vacancies are synthesized via lepidocrocite‐type titanate delamination and Pt single atoms are selectively trapped within these vacancies through a simple immersion process. The resulting Pt‐decorated nanosheets exhibit superior photocatalytic hydrogen evolution performance, outperforming both Pt nanoparticle‐loaded nanosheets and benchmarked Pt single‐atom catalysts on P25. Crucially, Pt atoms anchored at Ti^4+^ vacancies display remarkable resistance to light‐induced agglomeration, a key limitation of conventional single‐atom photocatalysts. Density functional theory calculations reveal that Pt incorporation into Ti^4+^ vacancies is highly thermodynamically favorable and optimizes hydrogen adsorption energetics for enhanced catalytic activity. This work highlights the critical role of cation defect engineering in stabilizing single‐atom co‐catalysts and advancing the efficiency and durability of photocatalytic hydrogen evolution.

## Graphical abstract

Pt single atoms are anchored in cation vacancies of Ti‐deficient TiO₂ nanosheets, offering exceptional stability against light‐induced agglomeration. These well‐trapped Pt atoms enable efficient and durable photocatalytic H_2_ evolution, outperforming Pt nanoparticles and surface‐bound single atoms on conventional TiO_2_. Combined experimental and computational studies highlight cation defect engineering as a powerful approach for stabilizing single atoms and enhancing photocatalytic performance.

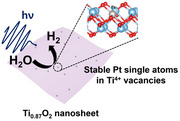

## 1 Introduction

Single‐atom (SA) catalysts have gathered significant attention in various fields of catalysis, such as heterogeneous catalysis,^[^ ^1^ ^]^ electrocatalysis,^[^ ^2^ ^]^ and recently also photocatalysis.^[^ ^3^, ^4^ ^]^ Semiconductors are commonly employed in conventional photocatalysis, as they can produce excited charge carriers (electron–hole pairs) when exposed to light with energy greater than their bandgap. These photoexcited carriers can then migrate to the semiconductor surface to trigger desired redox reactions, such as hydrogen evolution reactions. However, many semiconductors face challenges due to the slow charge‐transfer kinetics from the semiconductor conduction band to water or H^+^.^[^ ^5^ ^]^ To mitigate this, noble metal co‐catalysts such as Pt are often introduced to enhance the charge‐transfer and boost photocatalytic efficiency.^[^ ^6^, ^7^, ^8^, ^9^ ^]^ Considering the economic perspectives of the noble metal, extensive efforts have been dedicated to SA co‐catalysts because of their maximum surface‐to‐volume ratio and optimal atom utilization.^[^ ^10^, ^11^, ^12^, ^13^, ^14^, ^15^ ^]^ In addition, SA co‐catalysts can exhibit strong and dynamic interactions with the substrate, which may go beyond downscaling laws and can result in remarkable activity^[^ ^16^ ^]^.

Unlike other catalytic processes (e.g., thermal catalysis or electrocatalysis), it should be noted that only a comparably low loading density is needed in photocatalysis under typical light illumination conditions. In recent studies, an SA density ≈10^5^ atoms µm^−2^ was found to be sufficient ‐under common illumination conditions‐ to derive a maximized co‐catalytic effect and thus a maximum light utilization for the benchmark semiconductor, anatase TiO_2_.^[^ ^17^ ^]^ Nevertheless, SA co‐catalysts are highly prone to thermal‐induced and light‐induced agglomeration. For photocatalysis, it is therefore important to stabilize the isolated atoms and maintain the active sites to exploit the SA co‐catalysts with low, but sufficient density. Various anchoring methods have been developed for the synthesis of SAs/TiO_2_ catalysts,^[^ ^10^ ^]^ but the most widely used strategy for capturing SAs on the TiO_2_ surface involves trapping at defect sites, mostly oxygen vacancies.^[^ ^12^, ^13^, ^14^, ^15^, ^18^, ^19^, ^20^, ^21^, ^22^ ^]^ However, these SAs are still vulnerable to substantial agglomeration into nanosized particles by light‐induced activation and surface diffusion, which can deteriorate the long‐term photocatalytic performance.^[^ ^22^, ^23^ ^]^ Despite the inevitable need to establish both active and stable SA configurations, it is a great challenge to synthesize SAs/TiO_2_ photocatalysts that simultaneously exhibit high activity and resistance to agglomeration.

**Figure 1:**
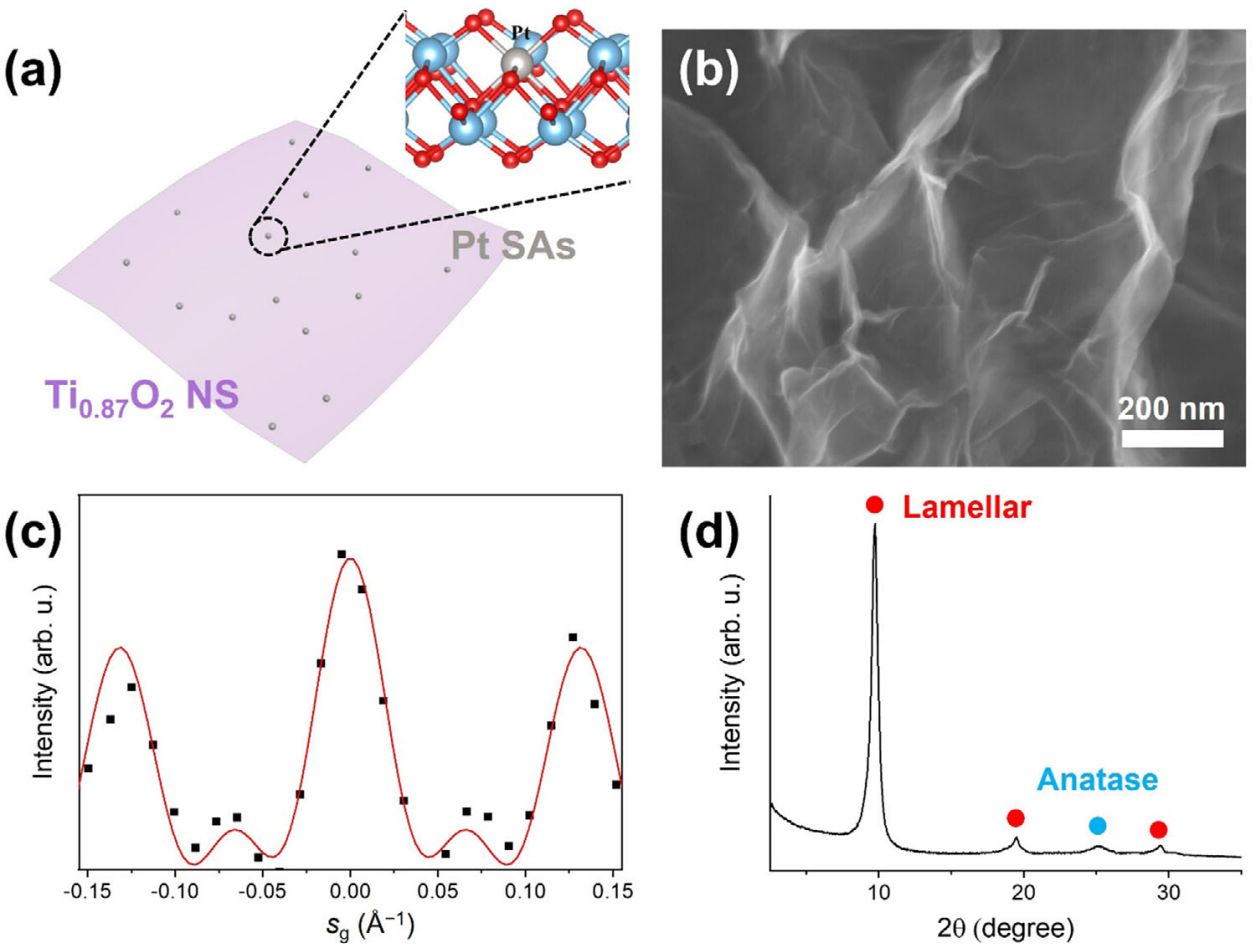
a) Schematic illustration of Pt SAs deposition in cation vacancies of Ti_0.87_O_2_ NSs. b) SEM image, c) rocking curve of TEM dark‐field intensity, and d) XRD pattern of Ti_0.87_O_2_ NSs. The positions of the anatase reference were taken from the ICDD database.

In the present work, we synthesize Ti‐deficient TiO_2_ nanosheets (Ti_0.87_O_2_ NSs) that can trap Pt SAs in their cation vacancies and thus act as efficient hosts for activating and stabilizing Pt SAs in photocatalytic hydrogen evolution reactions. Pt SAs capturing in the cation vacancies can be achieved by a straightforward dark deposition approach (**Figure** **1**a). The Ti_0.87_O_2_ NSs with Pt SAs exhibit a prominent photocatalytic activity, outperforming Pt SAs‐decorated P25 and Pt nanoparticles (NPs)‐deposited Ti_0.87_O_2_ NSs. Most remarkably, experimental and theoretical works find that the Ti^4+^ vacancies on the Ti_0.87_O_2_ NSs surface can effectively stabilize the Pt SAs against the light‐induced agglomeration – while Pt SAs stabilized on anion vacancies show agglomeration effects in minutes,^[^ ^22^, ^23^ ^]^ the cation vacancy reveals stability without any noticeable change after light irradiation. This work thus not only provides a tool for the remarkable stabilization of Pt SAs on TiO_2_ but also shows the feasibility of a fitting vacancy in advancing SA catalysis.

## 2 Results and Discussion

Ti_0.87_O_2_ NSs with Ti deficiencies were synthesized by protonating and osmotically delaminating lepidocrocite‐type titanate layers (K_0.8_Ti_1.73_Li_0.27_O_4_),^[^ ^24^, ^25^, ^26^ ^]^ as detailed in the Supporting Information. The composition of the NSs was determined using inductively coupled plasma optical emission spectroscopy (ICP‐OES).^[^ ^27^, ^28^ ^]^ Scanning electron microscopy (SEM) and transmission electron microscopy (TEM) images (Figure 1b; Figure S1, Supporting Information) reveal the atomically thin, sheet‐like morphology with a side length reaching several micrometers. The thickness of the NSs was measured through a rocking curve analysis, using a tilt series of dark‐field image mean intensity (Figure 1c).^[^ ^29^ ^]^ The derived rocking curve (red line) indicates that the Ti_0.87_O_2_ NSs possess a thickness of 2.25 nm, equivalent to a trilayer of TiO_2_ sheet with [010] orientation.^[^ ^24^, ^26^ ^]^ X‐ray diffraction (XRD) pattern (Figure 1d) further corroborates the sheet‐like structure, exhibiting a lamellar phase with a d‐spacing of 0.91 nm, along with a characteristic anatase peak at 25.3°. Additional peaks at 37.7°, 47.9°, 54.1°, 55.8°, 62.7°, 67.9°, 69.5°, and 75.1° (Figure S2, Supporting Information) are consistent with the anatase phase (ICDD 01‐071‐1166). Please note the lamellar d‐spacing of 0.91 nm deviates from the individual layer thickness of 0.75 nm due to water intercalation in between the NS layers^[^ ^24^, ^27^ ^]^ stemming from residual humidity during the XRD measurement as compared to the vacuum within the TEM.

To evaluate the bandgap of the Ti_0.87_O_2_ NSs, a Tauc plot is depicted in Figure S3a (Supporting Information) using data from transient photocurrent (inset of Figure S3a, Supporting Information) and incident‐photon‐to‐current efficiency (IPCE) measurements. Accordingly, a bandgap of *E_g_* ≈ 3.7 eV, larger than that of typical anatase TiO_2_ (3.2 eV),^[^ ^30^ ^]^ can be determined, which is due to quantum confinement in the ultrathin nature of the Ti_0.87_O_2_ NSs.^[^ ^31^, ^32^, ^33^ ^]^ The bandgap could further be confirmed to be ≈3.7 eV by diffuse reflectance spectroscopy (Figure S3b,c, Supporting Information). Transient photocurrents are recorded at various potentials and the corresponding voltage‐photocurrent plots display typical p‐type semiconductor behavior, with cathodic photocurrents increasing as the potential becomes more negative (Figure S3d, Supporting Information). The p‐type conductivity of the Ti_0.87_O_2_ NSs is also verified by their negative slope observed in Mott–Schottky plots (Figure S4, Supporting Information, the acceptor density and flat‐band potential are estimated to be 1.05 × 10^19^ cm^−3^ and 3.1 V versus Ag/AgCl, respectively). The p‐type characteristics reflect the presence of Ti^4+^ vacancies within the NSs since oxygen atoms near cation vacancies can attract relatively more electrons from surrounding Ti atoms, creating acceptor‐type defect states above the TiO_2_ valence band maximum^[^ ^34^, ^35^, ^36^ ^]^.

**Figure 2:**
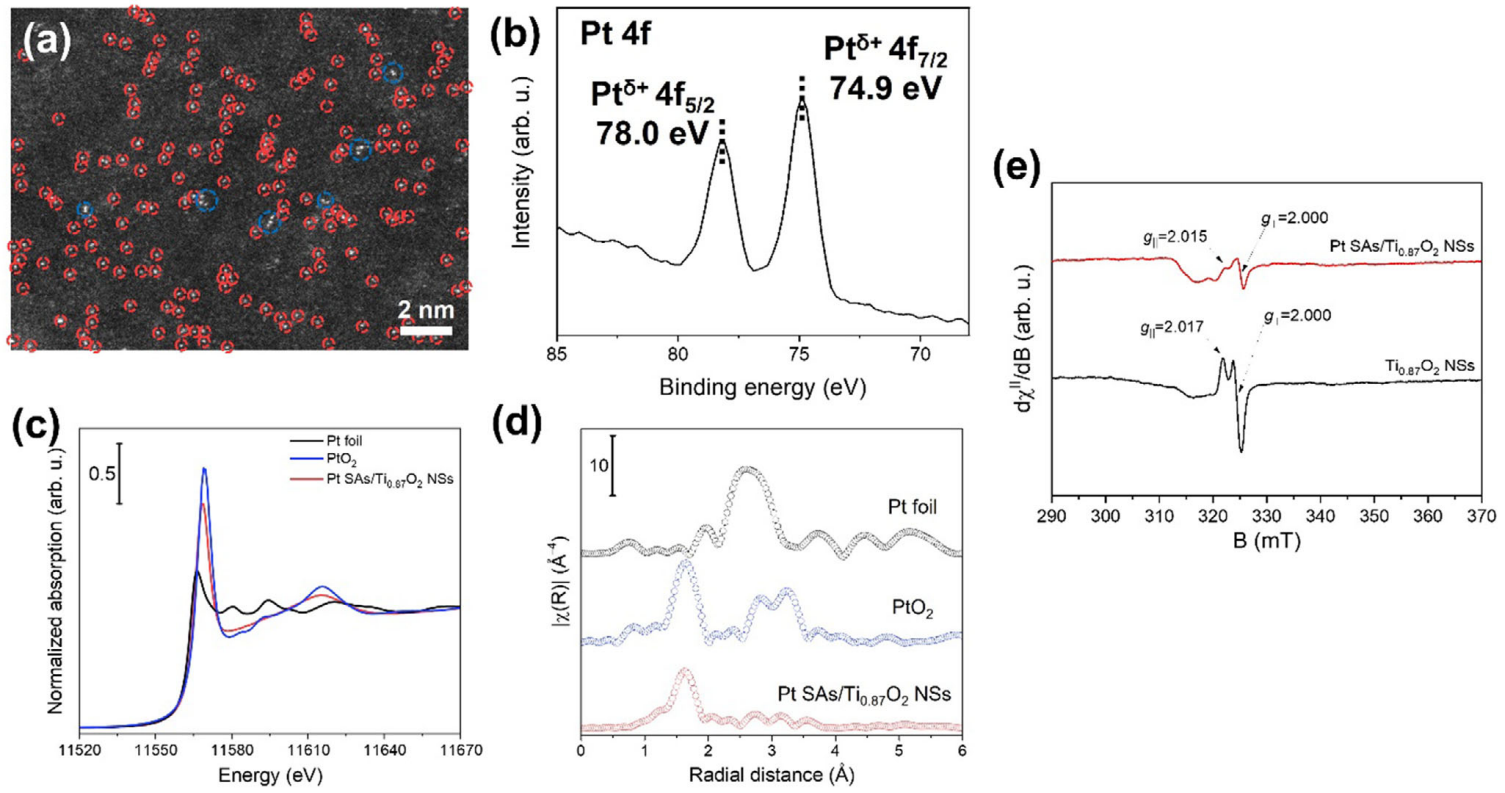
a) HAADF‐STEM image with highlighted SAs (red circles) and rafts (blue circles), b) Pt 4f XPS spectrum, c) Pt L_3_ edge normalized XANES spectra, and d) Fourier transform of k^3^‐weighted EXAFS spectra (R‐space) of Pt SAs/Ti_0.87_O_2_ NSs obtained with 0.5 mm H_2_PtCl_6_ solution. e) EPR spectra of Ti_0.87_O_2_ NSs and Pt SAs/Ti_0.87_O_2_ NSs.

To explore if the Ti^4+^ vacancies may provide capturing sites for Pt SAs, we exposed the sheets to a dilute platinic acid solution (0.5 mm H_2_PtCl_6_)‐ following a simple immersion (dark deposition) approach^[^ ^12^, ^13^, ^14^, ^15^, ^21^, ^22^ ^]^ (details of the Pt SAs/Ti_0.87_O_2_ NSs synthesis are provided in the Supporting Information). A high‐resolution SEM image of the NSs after Pt solution exposure, shown in Figure S5 (Supporting Information), reveals no noticeable changes in morphology or evidence of Pt deposition as NPs. The successful deposition of Pt SAs is confirmed through high‐angle annular dark‐field scanning transmission electron microscopy (HAADF‐STEM). As seen in the HAADF‐STEM image in **Figure** **2**a, Pt SAs are uniformly dispersed across the Ti_0.87_O_2_ NSs surface, as individual SAs as well as some mildly agglomerated rafts. From multiple HAADF‐STEM images of the Pt SAs/Ti_0.87_O_2_ NSs, an SA density of 7.5 × 10^5^ µm^−2^ can be estimated.

To investigate the oxidation state of the decorated Pt species, X‐ray photoelectron spectroscopy (XPS) was performed for the Pt SAs/Ti_0.87_O_2_ NSs. In the Pt 4f region, Figure 2b presents a doublet peak at 74.9 eV (Pt 4f_7/2_) and 78.0 eV (Pt 4f_5/2_), corresponding to a Pt^δ+^ species with a formal charge of approximately δ ≈ 4. This Pt 4f peak position deviates from the typical results for Pt SAs bound in an O‐coordinated configuration by dark deposition method, where Pt species are reduced by Ti^3+^ at oxygen vacancies via a galvanic replacement reaction and then deposited on the TiO_2_ surface.^[^ ^21^ ^]^ We suggest that the Pt SAs in the Ti_0.87_O_2_ NSs are captured through trapping within the cation vacancies.

X‐ray absorption near‐edge spectra (XANES) and extended X‐ray absorption fine structure (EXAFS) measurements were conducted to additionally explore the coordination environment of Pt species in Pt SAs/Ti_0.87_O_2_ NSs. The normalized XANES spectrum at the Pt L_3_‐edge (11 564 eV) indicates that Pt‐decorated NSs contain positively charged Pt centers (Figure 2c). The Fourier‐transformed radial distribution function (without phase correction) based on the k^3^‐weighted EXAFS spectrum reveals a primary feature at ≈1.6 Å attributed to Pt─O coordination (Figure 2d), suggesting that Pt species is present in a highly dispersed state without any metallic Pt─Pt bonding.

To further elucidate the defect structure of the NSs containing Ti^4+^ vacancies and their interplay with Pt SAs, electron paramagnetic resonance (EPR) studies were carried out. EPR spectrum of pristine Ti_0.87_O_2_ NSs recorded in dark conditions and in powder form, shown in Figure 2e (black line), exhibit strong and well‐resolved axial resonant line at g_||_ = 2.017 and g_⊥_ = 2.000, consistent with spin containing oxygen‐based center (Ti^4+^–O^•^ or/and Ti^4+^–O–O^•^). The strong and resolved resonance spectrum indicates that these spin systems remain highly localized, with negligible dipolar interactions occurring among spin defects,^[^ ^37^ ^]^ despite the introduction of lattice disorder brought by cation vacancies. Furthermore, no signals associated with Ti^3+^ centers can be detected.^[^ ^38^ ^]^ After Pt SA deposition, the EPR spectrum recorded in dark conditions of Pt SAs/Ti_0.87_O_2_ NSs shows some changes in the resonance signal (Figure 2e red line). Although the estimated g‐tensor parameters of the oxygen‐based radicals in Pt SAs/Ti_0.87_O_2_ NSs follow closely those determined in Ti_0.87_O_2_ NSs, giving here values at g_||_ = 2.015 and g_⊥_ = 2.000, the signals are both weaker and broader. This effect indicates that in the case of Pt SAs/Ti_0.87_O_2_ NSs, there is an increased dipolar interaction among spin‐defects, thus it points toward dense h^+^ spin clustering. The phenomenon mirrors subtle alterations in the TiO_2_ lattice due to the possible substitution of Ti with Pt (^2+^/^4+^) cations.

**Figure 3:**
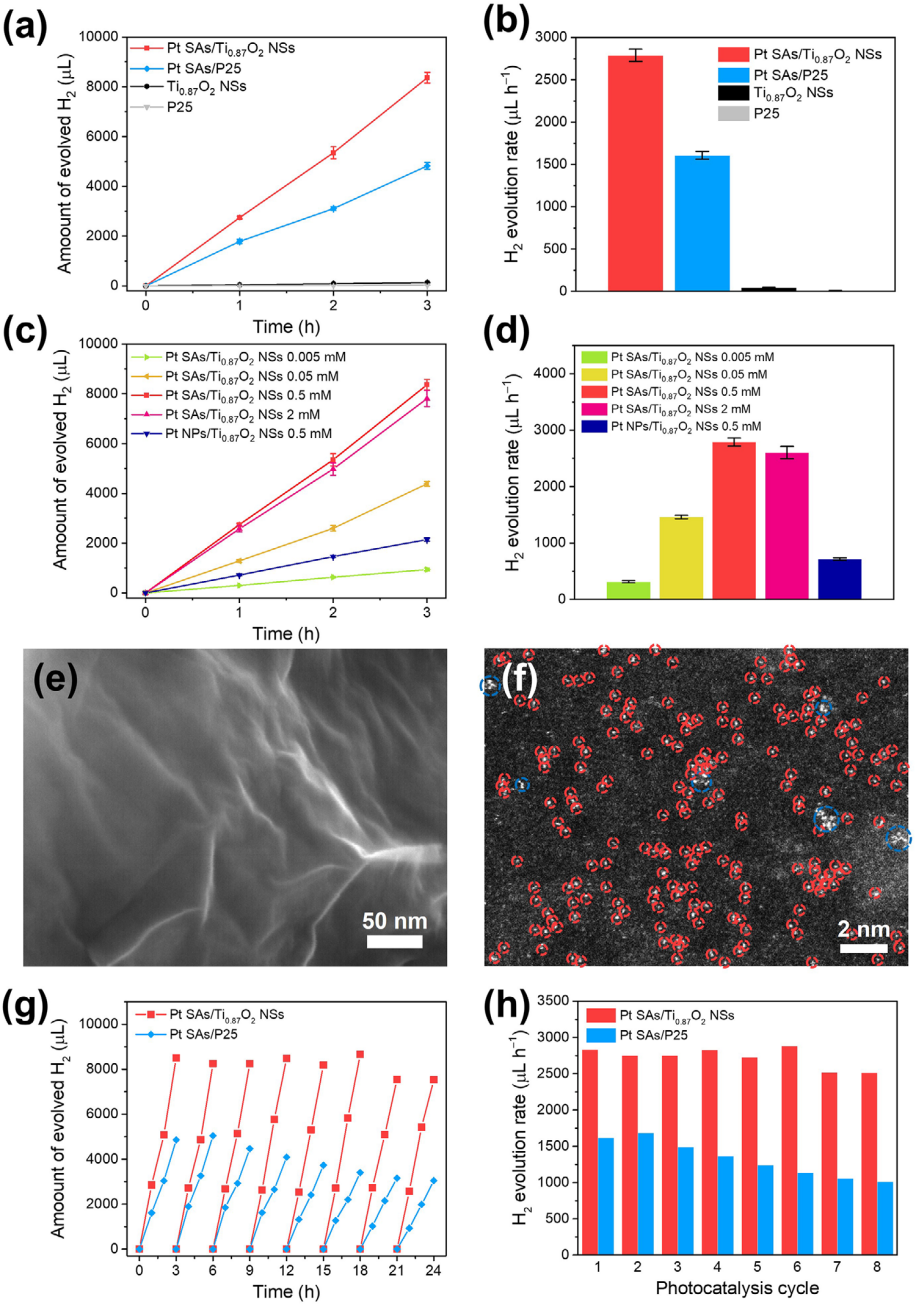
a) Amounts of hydrogen evolved and b) corresponding hydrogen evolution rate of Pt SAs/Ti_0.87_O_2_ NSs (0.5 mm), Ti_0.87_O_2_ NSs, Pt SAs/P25 (0.5 mm), and P25 under 275 nm LED irradiation. c,d) Photocatalytic hydrogen performance by Pt SAs/Ti_0.87_O_2_ NSs with different Pt precursor concentrations and Pt NPs/Ti_0.87_O_2_ NSs. e) SEM image and f) HAADF‐STEM image with highlighted SAs (red circles) and rafts (blue circles) of Pt SAs/Ti_0.87_O_2_ NSs after photocatalysis. g) Amounts of hydrogen evolved and h) corresponding hydrogen evolution rate of Pt SAs/Ti_0.87_O_2_ NSs and Pt SAs/P25 during the long‐term recyclability tests.

The photocatalytic performance for hydrogen evolution reaction of these Pt SAs‐decorated NSs was investigated under 275 nm LED illumination (10 mW cm^−2^). Please note that 275 nm LED was employed to ensure sufficient excitation beyond the bandgap of the NSs and demonstrate the concept of anchoring of Pt SAs at the cation vacancies. The performance was compared with those of Pt SAs/P25, neat Ti_0.87_O_2_ NSs, and P25. The amounts of H_2_ evolved during photocatalysis and the corresponding hydrogen evolution rates are summarized in **Figure** **3**a,b, respectively. Evidently, Pt SAs/Ti_0.87_O_2_ NSs exhibit the highest activity, with a remarkable H_2_ production rate of 2789 µL h^−1^ (1395 mL h^−1^ g^−1^). While the bare Ti_0.87_O_2_ NSs and P25 display a minor amount of H_2_ generation (Figure S6, Supporting Information), the deposition of Pt SAs clearly leads to a significant increase in the photocatalytic performance. Moreover, we examine the influence of Pt SA concentration on the photocatalytic activity of the Ti_0.87_O_2_ NSs by varying the Pt SAs loading concentrations (0.59, 0.48, 0.25, and 0.10 at.%, determined by XPS measurements) using different precursor concentrations (2, 0.5, 0.05, and 0.005 mm). Figure 3c shows the amounts of H_2_ produced by the catalysts over 3 h while the corresponding H_2_ production rates are illustrated in Figure 3d. The results indicate that the photocatalytic performance improves with increasing Pt loading up to 0.48 at.% (deposition with 0.5 mm H_2_PtCl_6_), but further increasing Pt loading does not provide a further benefit – the photocatalytic activity has reached saturation at a Pt loading of 0.48 at.% (7.5 × 10^5^ µm^−2^). Clearly, the Pt SAs/Ti_0.87_O_2_ NSs with an optimized loading outperform Pt NPs on the NSs (Figure S7, Supporting Information, even when loaded with a higher Pt concentration of 0.60 at.%).

Many photocatalytic systems that are co‐catalyzed by Pt SAs, such as oxygen‐coordinated Pt^δ+^ on TiO_2_, generally show a rapid and significant agglomeration into bigger NPs as soon as they are illuminated.^[^ ^22^, ^23^ ^]^ Within minutes of illumination, metallic Pt NPs are typically found.^[^ ^22^ ^]^ Indeed, Pt NPs can also be observed in the SEM image of Pt SAs/P25 after the photocatalysis (Figure S8, Supporting Information). This instability of Pt SAs poses a major problem for their continuous utilization. However, Pt SAs captured by cation vacancies display a distinctly more robust behavior. Figure 3e shows the SEM image of the Pt SAs/Ti_0.87_O_2_ NSs after the illumination experiment, evidently no light‐induced agglomeration of the Pt SAs can be seen. The HAADF‐STEM image shown in Figure 3f further confirms that Pt SAs on the NSs retain their SA configuration remarkably well, even after the photocatalytic experiments. In addition, the oxidation state of Pt species remains largely unchanged in the Pt SAs/Ti_0.87_O_2_ NSs, whereas Pt SAs on P25 are rapidly reduced to the metallic state upon minute‐scale light irradiation (Figure S9, Supporting Information). Given the exceptional persistence of Pt SA configuration on the Ti_0.87_O_2_ NSs, we evaluated the H_2_ evolution performance of the Pt SAs/Ti_0.87_O_2_ NSs in long‐term recyclability tests and compared with that of the Pt SAs/P25 (Figure 3g,h). Notably, Pt SAs‐decorated NSs maintain their excellent performance throughout the eight consecutive runs (≈10% of activity decrease after 24 h). In contrast, Pt SAs loaded on P25 exhibit a stark activity decay (≈40% of activity decrease after 24 h) over the photocatalysis cycles.

**Figure 4:**
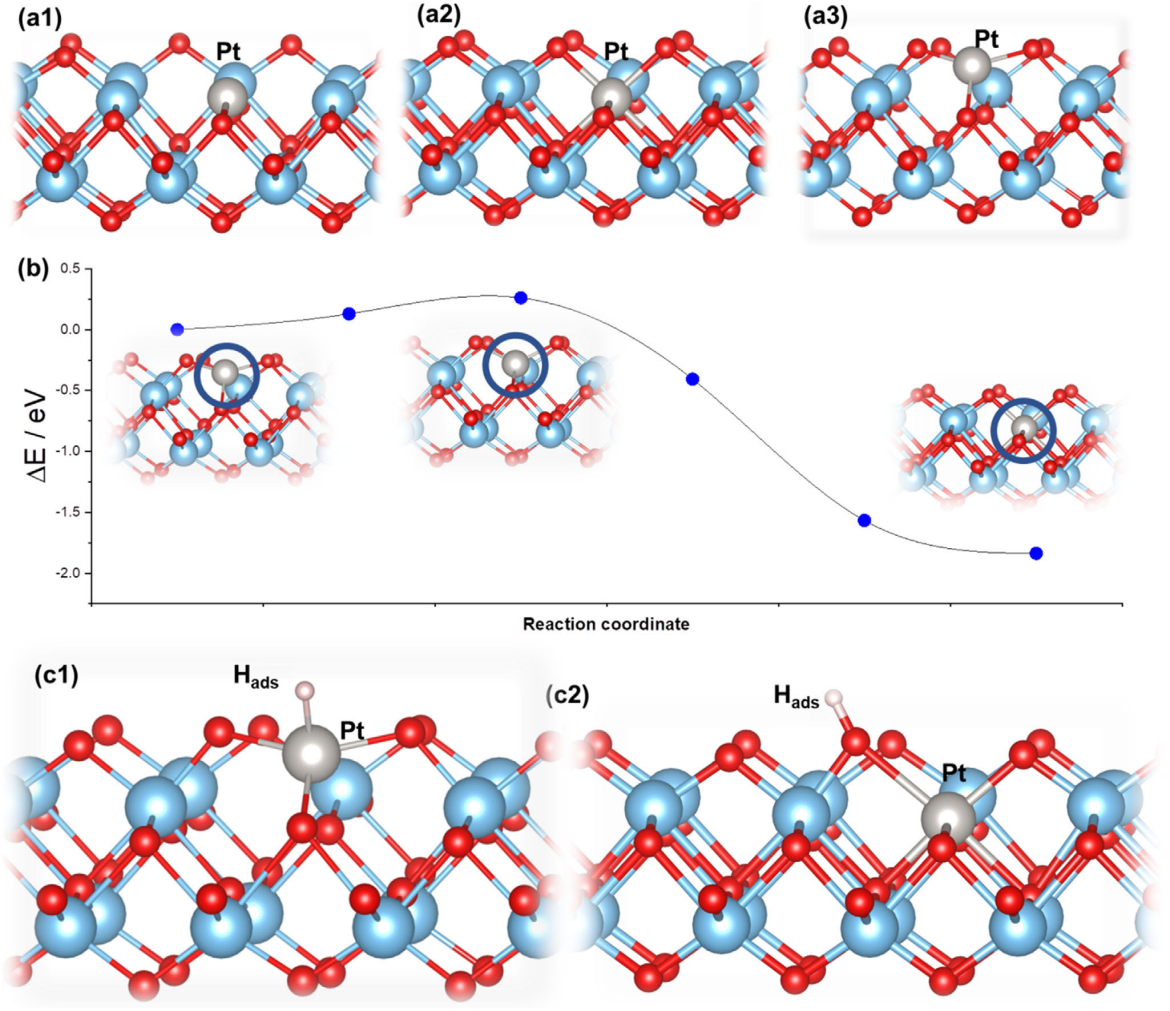
DFT models of Pt SA in a1) TiO_2_ vacancy (Schottky defect), a2) Ti^4+^ vacancy, and a3) surface‐bound Pt SA on titania NS. b) Kinetic barrier for Pt SA diffusion into the Ti^4+^ vacancy. c1) The optimized structure of H_ads_ formation on the surface‐bound Pt SA (*|*Δ*G*_H_ *|* = 0.13 eV) and c2) on the Pt‐bonded oxygen surface site for Pt SA in Ti^4+^ vacancy (*|*Δ*G*_H_ *|* = 0.14 eV).

Density functional theory (DFT) calculations further suggest a very strong incorporation of Pt atoms in the Ti^4+^ vacancy. Compared to Pt incorporation into a TiO_2_ vacancy (Schottky defect, **Figure** **4**a, 1), which deliberates −2.78 eV, the binding of Pt SA into the Ti^4+^ vacancy (Figure 4a2), resulting in the formal Pt^4+^ SA, exhibits a significantly stronger binding energy of −11.84 eV. This energy is more than twice as high as the cohesive energy of bulk platinum (5.84 eV),^[^ ^39^ ^]^ suggesting that the SA state in Ti^4+^ vacancy is more favorable than forming Pt agglomerates.^[^ ^40^ ^]^ Molecular dynamics calculations confirmed that both Ti^4+^ vacancy and Pt SA incorporated into the Ti^4+^ vacancy are stable at room temperature (Figure S10, Supporting Information). Moreover, we found charge localization and spin appearance at O atoms around the Ti^4+^ vacancy, without indications of Ti^3+^ centers, in line with the EPR results (Figure S11, Supporting Information). The incorporation of Pt SA into the Ti^4+^ vacancy was further studied using climbing image nudged elastic band calculations. The most stable configuration of Pt SA on the surface is a three‐coordinated coordinated position in the vicinity of the Ti^4+^ vacancy (Figure 4a3). Pt binding energy at this site is also very high (10.00 eV), suggesting that this configuration is also thermodynamically favored. However, the barrier for Pt SA migration into the Ti^4+^ vacancy was found to be 0.26 eV (Figure 4b), implying that migration can take place under room‐temperature conditions.

Considering the high photocatalytic H_2_ generation of Pt SAs/Ti_0.87_O_2_ NSs, we additionally investigated how strongly H atoms are bound to the surface‐bound Pt SA and Pt SA captured in Ti^4+^ vacancy. The surface‐bound Pt SA binds the H atom with Gibbs free energy for H_ads_ formation (*|*Δ*G*_H_ *|*) of 0.13 eV (Figure 4c1). On the other hand, Pt SA embedded in the Ti^4+^ vacancy is screened by the surface O atoms, but the presence of Pt species in the cation vacancy enables the H atom adsorption on the adjacent surface O atoms bonded to the Pt SA (Figure 4c2), giving the *|*Δ*G*_H_ *|* = 0.14 eV, close to that of surface‐bound Pt SA and Pt(111) (0.09 eV).^[^ ^41^ ^]^ Given that direct H atom adsorption on Pt SA weakens the Pt binding to the TiO_2_ surface and makes it more mobile across the surface,^[^ ^22^ ^]^ these findings further suggest that Pt SAs captured in the cation vacancies can exhibit enhanced durability even under photocatalytic H_2_ evolution condition, while still achieving high catalytic activity despite the absence of direct H adsorption.

The combination of experimental and theoretical studies clearly demonstrates that the ultrathin titania NSs with cation vacancies are effective substrates for Pt SAs not only in terms of photocatalytic activity for hydrogen evolution reaction, but also in stabilizing the isolated atoms – the coordination of Pt SAs with the Ti^4+^ vacancy provides significant resistance to the photoinduced agglomeration, resulting in the stable catalytic performance.

## 3 Conclusion

In conclusion, this work presents a novel strategy for stabilizing Pt SAs on TiO_2_ by leveraging cation vacancies in Ti‐deficient TiO_2_ NSs. We demonstrate that these vacancies provide exceptionally stable anchoring sites, preventing light‐induced agglomeration—an issue that typically undermines the long‐term performance of SA photocatalysts. The resulting Pt‐decorated TiO_2_ NSs exhibit outstanding photocatalytic hydrogen evolution activity, significantly surpassing Pt SA catalysts on conventional TiO_2_ substrates and Pt NP‐based systems. Experimental and theoretical investigations reveal that Pt atoms embedded in Ti^4+^ vacancies exhibit strong thermodynamic stability and optimal hydrogen adsorption energetics, driving efficient hydrogen evolution. In stark contrast to oxygen vacancy‐trapped SAs, which rapidly aggregate under illumination, Pt atoms in cation vacancies maintain their atomic dispersion after photocatalysis, ensuring sustained catalytic performance. These findings highlight the potential of defect engineering in tailoring semiconductor surfaces for SA catalysis, offering a robust and scalable approach to enhancing photocatalyst stability and efficiency. Beyond photocatalytic hydrogen production, this strategy may be extended to other catalytic systems requiring stable SA configurations, paving the way for much wider applications.

## 4 Experimental Section

### Chemicals and Materials

K_2_CO_3_ (Sigma–Aldrich, 99.99%), Li_2_CO_3_ (Mateck, ≥99.995%), TiO_2_ (rutile, Sigma–Aldrich, ≥99.9%), HCl (Carl Roth, 37%), tetrabutylammonium hydroxide (TBAOH, Sigma–Aldrich, 40 wt.% in water), P25 (Sigma–Aldrich, ≥99.5%), H_2_PtCl_6_ (Metakem, 40.17% metallic Pt weight concentration), Na_2_SO_4_ (Carl Roth, ≥99%), methanol (Carl Roth, 99.9%) were used as received without any purification. Aqueous solutions were prepared using highly purified water with a resistivity of 18.2 mΩ·cm.

### Synthesis of Ti_0.87_O_2_ NSs

The titania NSs were produced by delaminating the pristine layered K_0.8_Ti_1.73_Li_0.27_O_4_, following a method previously detailed in the literature that involves three distinct stages.^[^ ^24^, ^25^ ^]^ Initially, the pristine layered K_0.8_Ti_1.73_Li_0.27_O_4_ was synthesized through a solid‐state calcination process, using K_2_CO_3_, Li_2_CO_3_, and TiO_2_ (rutile) in a molar ratio of 0.4:0.14:1.73. The starting materials were ground in a ceramic mortar for 30 min, with the addition of 10 mL of acetone to facilitate uniform mixing of the powders. This mixture was subsequently placed in a platinum crucible and subjected to decarbonization at 800 °C for 1 h. After slowly cooling to room temperature, the sample was ground again for 30 min and compacted into pellets. The final step involved calcining the pellets at 1100 °C for a duration of 1 week in a platinum crucible. In the second stage, the protonated product, H_1.07_Ti_1.73_O_4_·H_2_O, was obtained by dispersing K_0.8_Ti_1.73_Li_0.27_O_4_ in a 1 m HCl solution and stirring under standard laboratory conditions. The ion exchange reaction was carried out over a period of two days. After 24 h, a decantation was conducted, followed by the addition of fresh 1 m HCl solution. Once the final reaction was completed, another decantation was performed. To eliminate the HCl solution, the protonated sample was extensively rinsed with water using centrifugation. At the last stage, the delamination of the protonated layered titanate compound was initiated by adding a TBAOH solution and shaking the mixture for 1 week, adjusting the TBAOH concentration to achieve a molar ratio of TBA^+^/H^+^  = 1. To eliminate any excess TBA⁺ ions that were adsorbed on the NS surface, the samples underwent thorough washing with water through centrifugation.

### Pt SAs and NPs Deposition

To deposit Pt SAs onto TiO_2_ samples (Ti_0.87_O_2_ NSs and P25), 15 mg of TiO_2_ powder was dispersed in 75 mL of water, and the mixture was purged with Ar for 15 min. Then, H_2_PtCl_6_ was added to achieve the target concentration of the Pt precursor. The solution was stirred for 1 h in the dark, and the resulting samples were washed by centrifugation at 12 000 rpm for 20 min. For the photodeposition of Pt NPs, 10 mg of the TiO_2_ was added to 10 mL of methanol aqueous solution (50 vol.%) and the resultant solution was purged with Ar for 15 min. Then, H_2_PtCl_6_ was added to the solution to make the overall Pt precursor concentration of 0.5 mm. UV irradiation (λ = 275 nm, power density = 10 mW cm^−2^) was conducted for 24 h and the samples were washed by centrifugation at 12 000 rpm for 20 min.

### Characterization

Chemical composition analyses were performed by ICP‐OES (Perkin Elmer Avio 200). A Hitachi S‐4800 scanning electron microscope was utilized to take SEM images, while HAADF‐STEM images were obtained using a high‐resolution transmission electron microscope (Spectra 200 C‐FEG, Thermo Fisher Scientific) with probe correction and a cold field emission gun (X‐CFEG). Rocking curves of dark‐field intensities from Ti_0.87_O_2_ NSs with {010} orientation were acquired to precisely determine the individual layer thickness as well as the number of layers of the investigated NS.^[^ ^29^ ^]^ A theoretical rocking curve, assuming an equilibrium interlayer distance of 0.75 nm,^[^ ^24^, ^26^ ^]^ was calculated within the kinematic diffraction theory utilizing the following Equation (1):

(1) $$I=A{\left(\frac{\sin\left(\pi \times N\times 0.75\;nm\times s\right)}{\sin\left(\pi \times 0.75nm\times s\right)}\right)}^{2}\times e^{-Bs^{2}}+C$$

where *A* is a scaling factor, *N* is the number of layers, *s* is the excitation error, *B* is a damping factor taking into account the angular dependence of the scattering factor, and *C* representing the constant background. The lamellar spacing can be directly read out by the inverse of the position of the first order maxima ≈± 0.133 Å^−1^, whereas the number of side maxima (N−1) gives access to the number of involved layers in analogy to scattering at an optical slit system. The XRD pattern was measured with an X‐ray diffractometer (X'pert Philips MPD equipped with a Panalytical X'celerator detector) utilizing graphite‐monochromatized Cu Kα radiation (λ = 0.15 406 nm). XPS analysis was performed using a PHI 5600 X‐ray photoelectron spectrometer (US) with monochromatic Al Kα radiation (1486.6 eV, 300 W) to investigate the chemical composition of the samples. X‐ray absorption spectra at the Pt L_3_‐edge (11 564 eV) were collected at the P65 beamline of the Deutsches Elektronen‐Synchrotron (DESY), Hamburg, Germany. A Si (111) double crystal monochromator was used to scan the incident energy. The energy calibration was performed using a reference platinum metal foil. For measuring the reference samples, pellets diluted with cellulose were used, and the measurements were conducted in transmission geometry. For ex situ measurements of the Pt SAs/Ti_0.87_O_2_ NSs, the catalyst was kept inside a plastic sample holder (5 mm path length) without dilution, and the measurements were conducted in fluorescence geometry using a Hitachi Vortex‐ME4 silicon drift detector. The processing of the XAS data (data reduction, alignment, normalization, background subtraction, Fourier transformation) was performed using the Athena code (version 0.9.26). EXAFS data was k^3^‐weighted and Fourier transformed in the range of 2–14 Å^−1^. EPR spectra were recorded on a JEOL JES‐X‐320 X‐band (≈9.14–9.17 GHz) spectrometer, equipped with a variable He temperature control setup (ES‐CT470). Measurements were conducted at 85 K, maintaining a quality factor (Q) above 6000 for comparability. High‐purity quartz tubes (Suprasil, Wilmad, ≤ 0.5 OD) thoroughly degassed under N_2_ atmosphere served as sample holders, and g‐value accuracy was determined using a Mn^2+^/MgO standard (JEOL standard). The microwave power was set at 1.0 mW to avoid power saturation effects. A modulation width of 1 mT and modulation frequency of 100 kHz were used, with EPR spectra recorded using a 30 ms time constant and 2 min sweep time across three accumulations to enhance the signal‐to‐noise ratio.

### Photoelectrochemical Analysis

Electrochemical and photoelectrochemical analyses, including IPCE, transient photocurrent response, and Mott–Schottky plot measurements, were conducted using a three‐electrode cell setup. In this configuration, the Ti_0.87_O_2_ NSs sample loaded on a carbon electrode was used as the working electrode, a Pt plate as the counter, and an Ag/AgCl electrode as the reference. The electrolyte was a 0.1 m Na_2_SO_4_ aqueous solution, purged with Ar. IPCE measurements were taken at a constant potential of −0.5 V versus Ag/AgCl, in an illumination wavelength range of 250–600 nm, using an Oriel 6365 150 W Xe‐lamp with an Oriel Cornerstone 7400 1/8 m monochromator as the light source. Transient photocurrent response was recorded at potentials from −0.1 to −0.6 V versus Ag/AgCl at a wavelength of 320 nm (power density = 0.15 mW cm^−2^), using the same light source as for IPCE, following this sequence: light off (10 s), light on (20 s), and light off (10 s). Mott–Schottky analysis was carried out at various frequencies with a potential perturbation of 10 mV.

### Photocatalysis

The photocatalytic hydrogen evolution reaction was carried out under UV LED illumination with a wavelength of 275 nm and a power density of 10 mW cm^−2^. In this experiment, 2.0 mg of catalyst was dispersed in 10 mL of a 50 vol.% methanol aqueous solution inside a quartz cell. To ensure anaerobic conditions, the solution was purged with Ar gas for 15 min before the illumination. The amounts of hydrogen generated were determined using gas chromatography (GCMS‐QO2010SE, SHIMADZU) with a thermal conductivity detector.

### DFT Calculation

The first‐principle DFT calculations were performed using the Vienna ab initio simulation code (VASP).^[^ ^42^, ^43^, ^44^ ^]^ The Generalized Gradient Approximation (GGA) in the parametrization by Perdew, Burk, and Ernzerhof^[^ ^45^ ^]^ combined with the projector augmented wave (PAW) method was used,^[^ ^46^ ^]^ combined with the DFT+*U* approach to treat electron localization,^[^ ^47^ ^]^ with *U* = 3.5 eV added to the Ti d states. The Ti‐deficient TiO_2_ NS models were adopted from the reference ^[^ ^48^ ^]^, with 16 Ti and 32 O atoms in the pristine form. Then Ti^4+^ or TiO_2_ vacancy was introduced in the NS. A cut‐off energy of 450 eV was used with the Monkhorst–Pack Γ‐centered 3 × 3 × 1 *k*‐point mesh, while optimization was run until the forces acting on all atoms were below 0.005 eV Å^−1^. Spin polarization was included in all the calculations. The Ti and H binding energies reported here were calculated with respect to isolated atoms and more negative values indicate stronger binding. The stability of the investigated models was probed using standard molecular dynamics at 298 K, with Nosé–Hoover thermostat.^[^ ^49^, ^50^ ^]^ The time step was set to 1 fs and the duration was 1000 fs. Migration of Pt SA into Ti^4+^ vacancy was studied using a climbing image nudged elastic band method.^[^ ^51^ ^]^

## Conflict of Interest

The authors declare no conflict of interest.

## Supporting information



Supporting Information

## Data Availability

The data that support the findings of this study are available from the corresponding author upon reasonable request.
