# Supplementary material for: Cation Vacancies in Ti‐Deficient TiO2 Nanosheets Enable Highly Stable Trapping of Pt Single Atoms for Persistent Photocatalytic Hydrogen Evolution
Source: Small. 2025 Jun 2;21(29):2502428. doi: 10.1002/smll.202502428 (PMC12288771; doi:10.1002/smll.202502428)
Supplement: Supplementary file 1 — Supporting Information [file SMLL-21-2502428-s001.docx]

Supporting information

**Cation Vacancies in Ti-Deficient TiO_2_ Nanosheets Enable Highly Stable Trapping of**

**Pt Single Atoms for Persistent Photocatalytic Hydrogen Evolution**

Hayoon Jung, ^†, a, b^ Gihoon Cha, ^†, c, d^ Hyesung Kim, ^a^ Johannes Will, ^e^ Xin Zhou, ^e^

Zdeněk Bad'ura, ^b, f^ Giorgio Zoppellaro, ^b, f^ Ana S. Dobrota, ^g^ Natalia V. Skorodumova, ^h^

Igor A. Pašti, ^g, i^ Bidyut Bikash Sarma, ^j^ Jochen Schmidt, ^k^ Erdmann Spiecker, ^e^ Josef Breu, ^d^ and Patrik Schmuki ^a, b *^

^a^ Department of Materials Science and Engineering, WW4-LKO, Friedrich-Alexander-Universität Erlangen-Nürnberg, Martensstraße 7, 91058 Erlangen, Germany

^b^ Regional Centre of Advanced Technologies and Materials, Czech Advanced Technology and Research Institute (CATRIN), Palacký University, Šlechtitelů 27, 78371 Olomouc, Czech Republic

^c^ Department for Correlative Microscopy and Materials Data, Fraunhofer Institute for Ceramic Technologies and Systems (IKTS), Äußere Nürnberger Straße 62, 91301 Forchheim, Germany

^d^ Department of Chemistry, Universität Bayreuth, Universitätsstraße 30, 95447 Bayreuth, Germany

^e^ Institute of Micro- and Nanostructure Research & Center for Nanoanalysis and Electron Microscopy (CENEM), IZNF, Friedrich-Alexander-Universität Erlangen-Nürnberg, Cauerstraße 3, 91058 Erlangen, Germany

^f^ Nanotechnology Centre, VŠB − Technical University of Ostrava, 17. listopadu 2172/15, 708 00 Ostrava-Poruba, Czech Republic

^g^ University of Belgrade – Faculty of Physical Chemistry, Studentski trg 12-16, 11000 Belgrade, Serbia

^h^ Applied Physics, Division of Materials Science, Department of Engineering Sciences and Mathematics, Luleå University of Technology, 971 87 Luleå, Sweden

^i^ Serbian Academy of Sciences and Art, Kneza Mihaila 35, 11000 Belgrade, Serbia

^j^ Laboratoire de Chimie de Coordination (LCC), CNRS, Université de Toulouse, INPT, UPR 8241, 205 route de Narbonne, 31077 Toulouse Cedex 4, France

^k^ Institute of Particle Technology, Friedrich-Alexander-Universität Erlangen-Nürnberg, Cauerstraße 4, 91058 Erlangen, Germany

[^†^] These authors contributed equally to this work.

E-mail: schmuki@ww.uni-erlangen.de

**
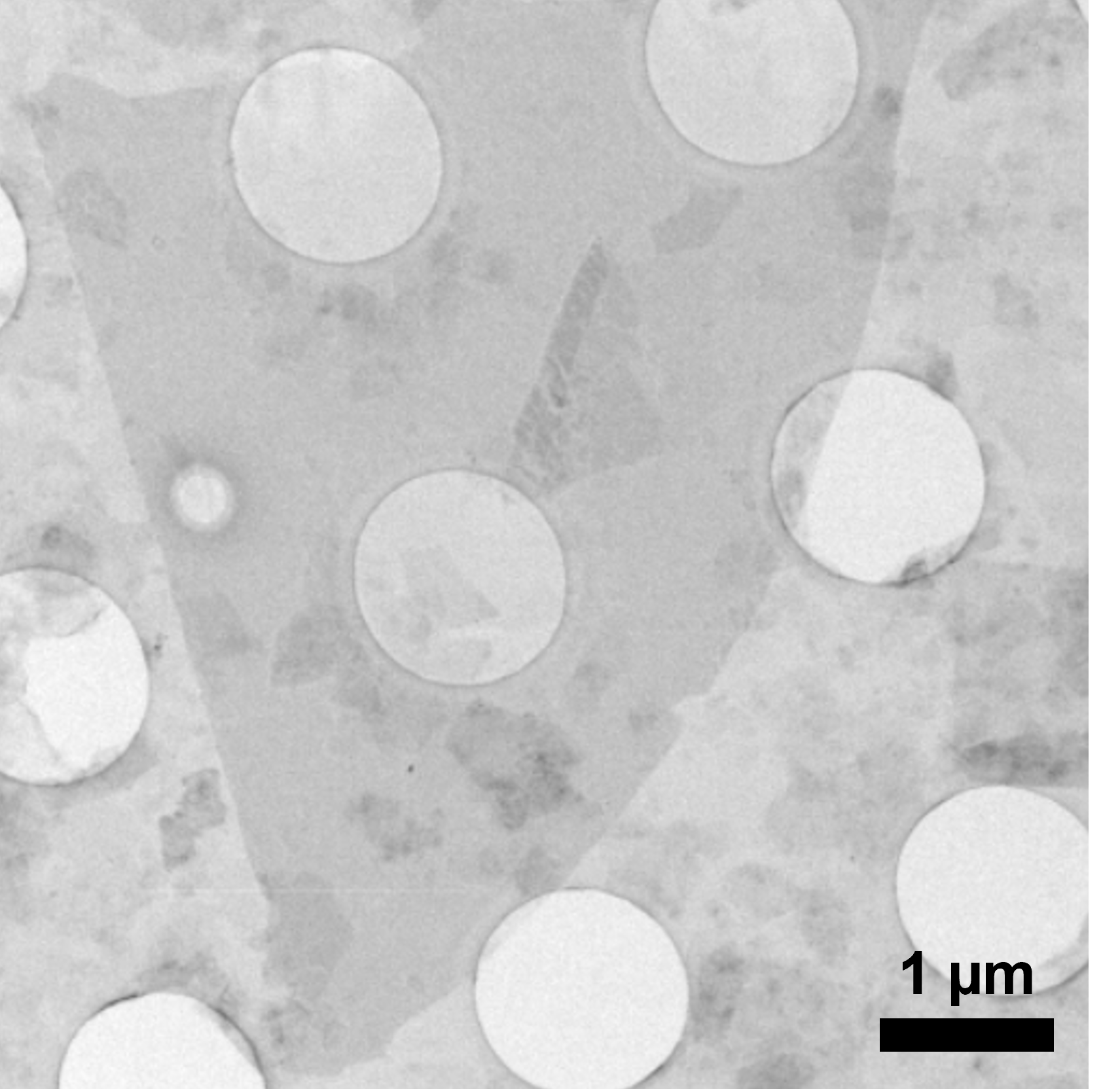
**

**Fig. S1.** TEM image of Ti_0.87_O_2_ NSs.

**
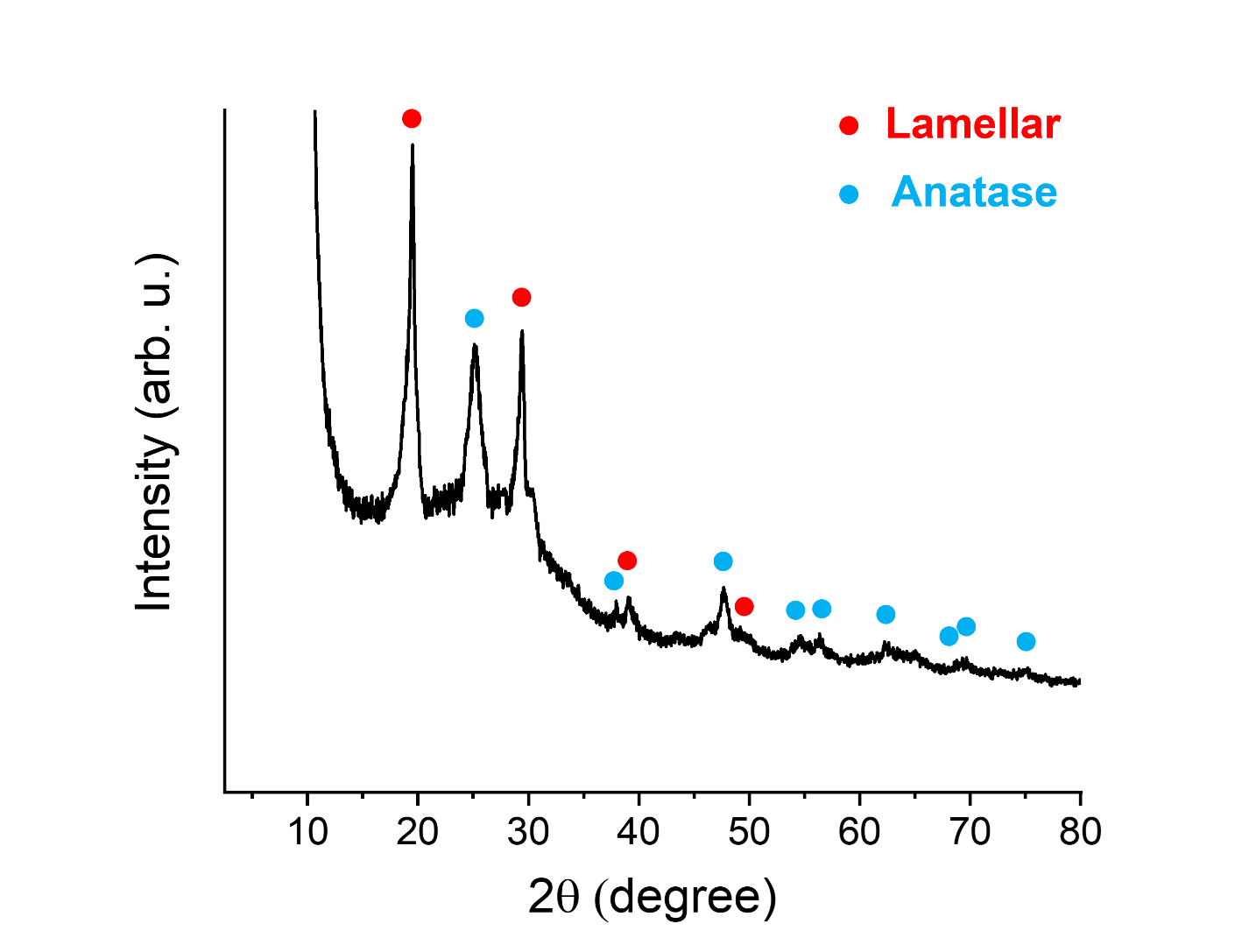
**

**Fig. S2.** Detailed XRD pattern of Ti_0.87_O_2_ NSs. The positions of the anatase reference were taken from the ICDD database.


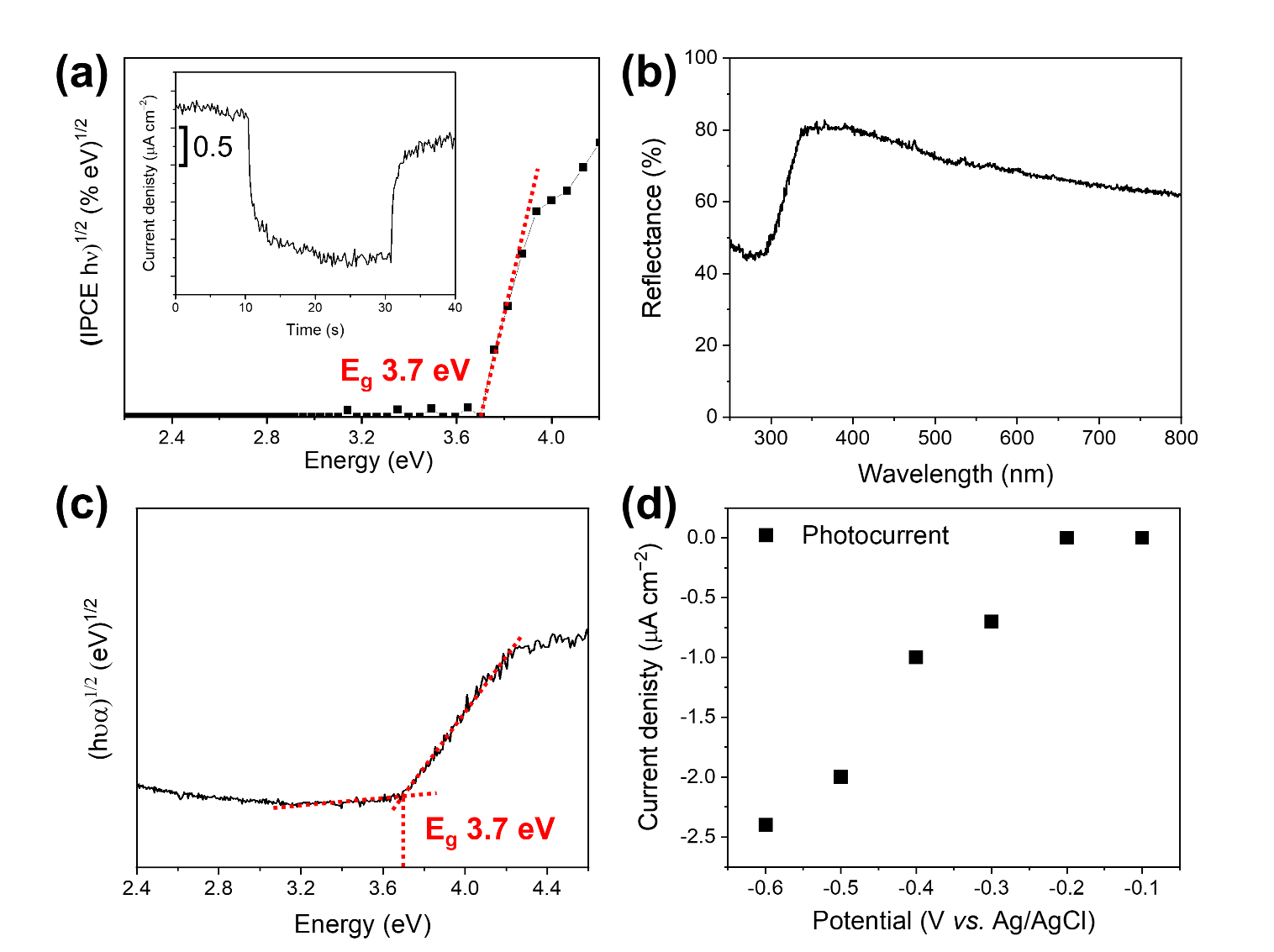


**Fig. S3.** (a) Tauc plot of Ti_0.87_O_2_ NSs by IPCE measurements (Inset: transient photocurrent curve at −0.5 V *vs.* Ag/AgCl under 320 nm monochromatic irradiation). (b) Diffuse reflectance spectroscopy and (c) corresponding Tauc plot of Ti_0.87_O_2_ NSs. (d) Photocurrents of Ti_0.87_O_2_ NSs at various potentials under 320 nm monochromatic irradiation.

**
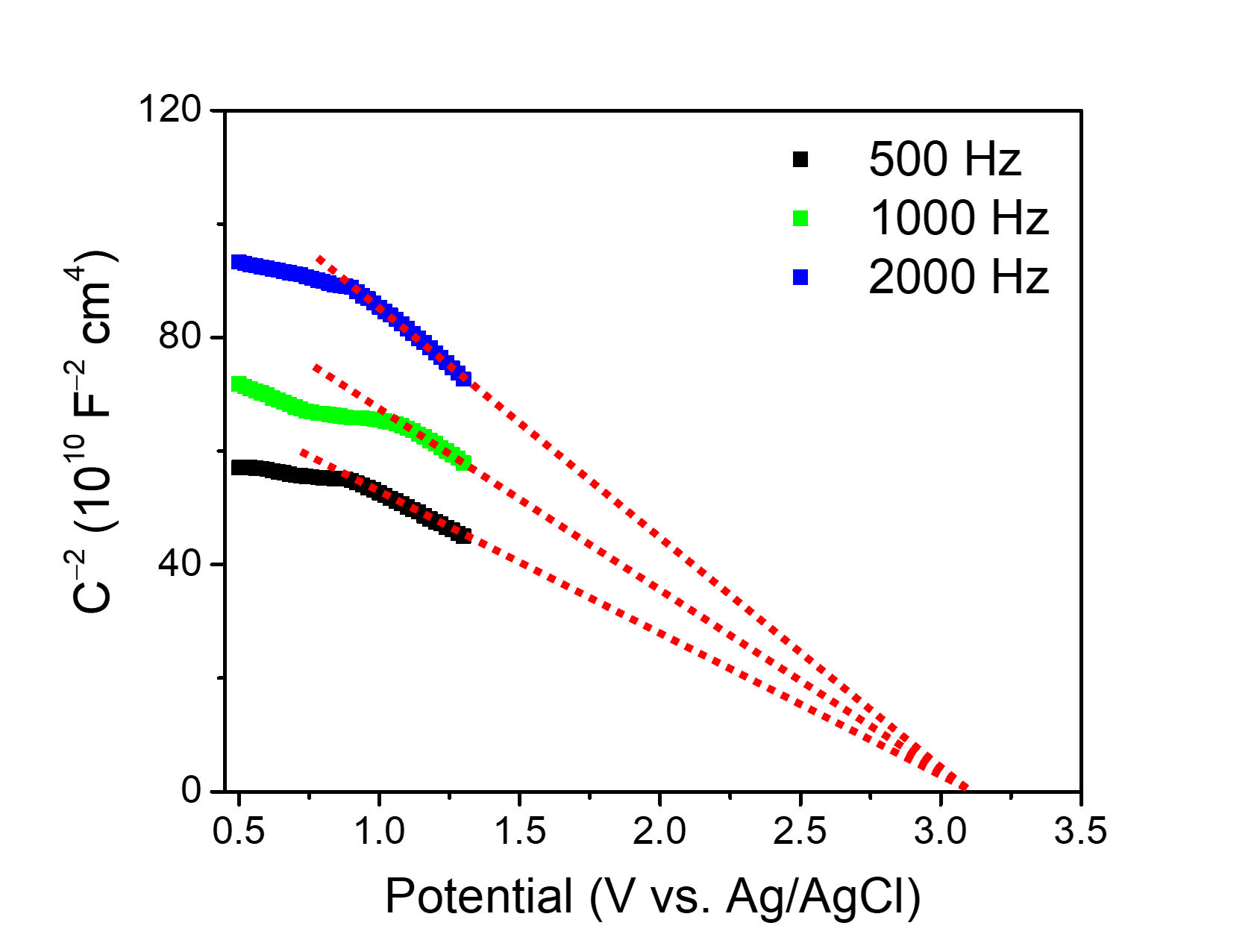
**

**Fig. S4.** Mott-Schottky plots of Ti_0.87_O_2_ NSs.

**
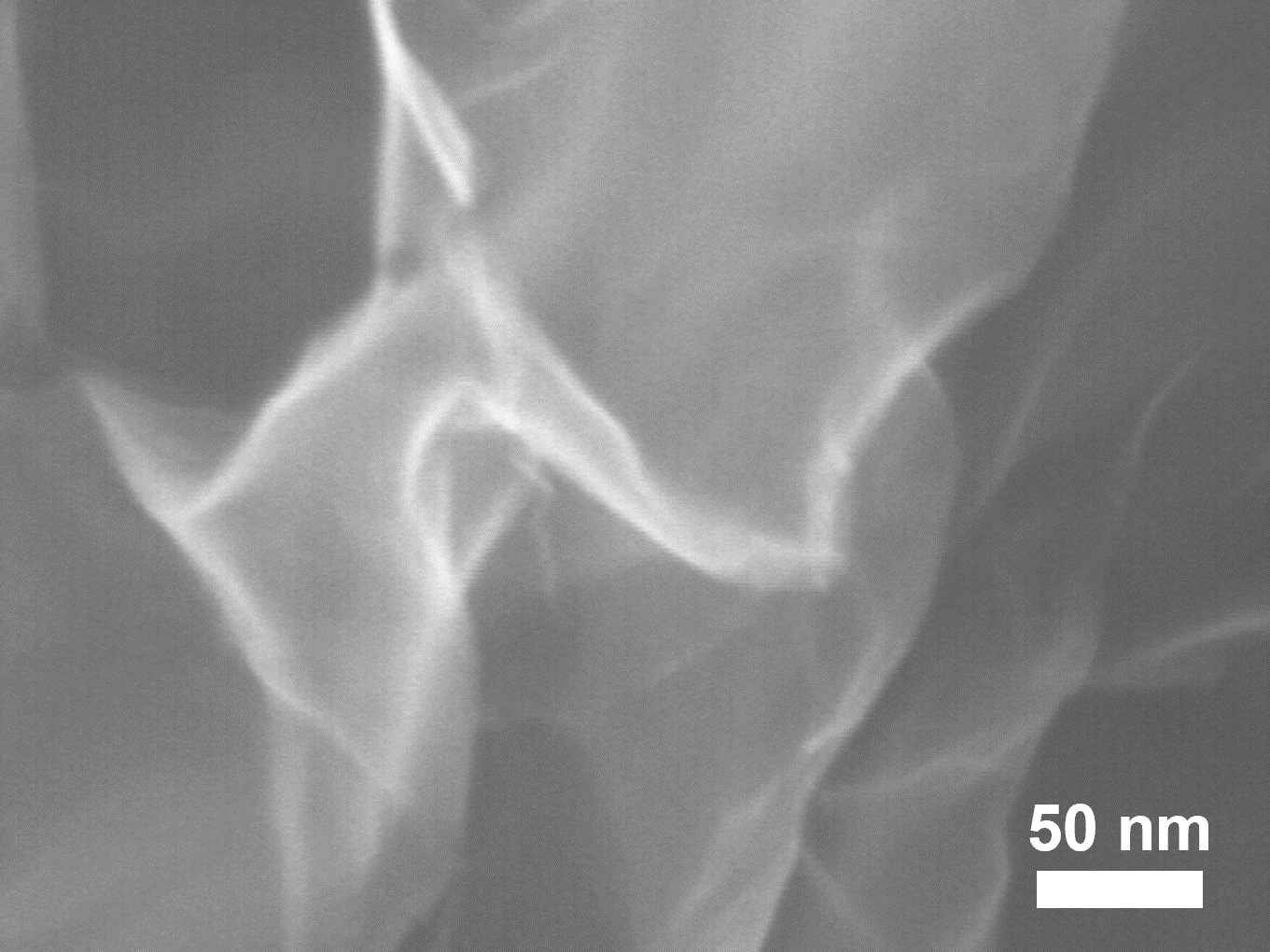
**

**Fig. S5.** SEM image of Pt SAs/Ti_0.87_O_2_ NSs.

**
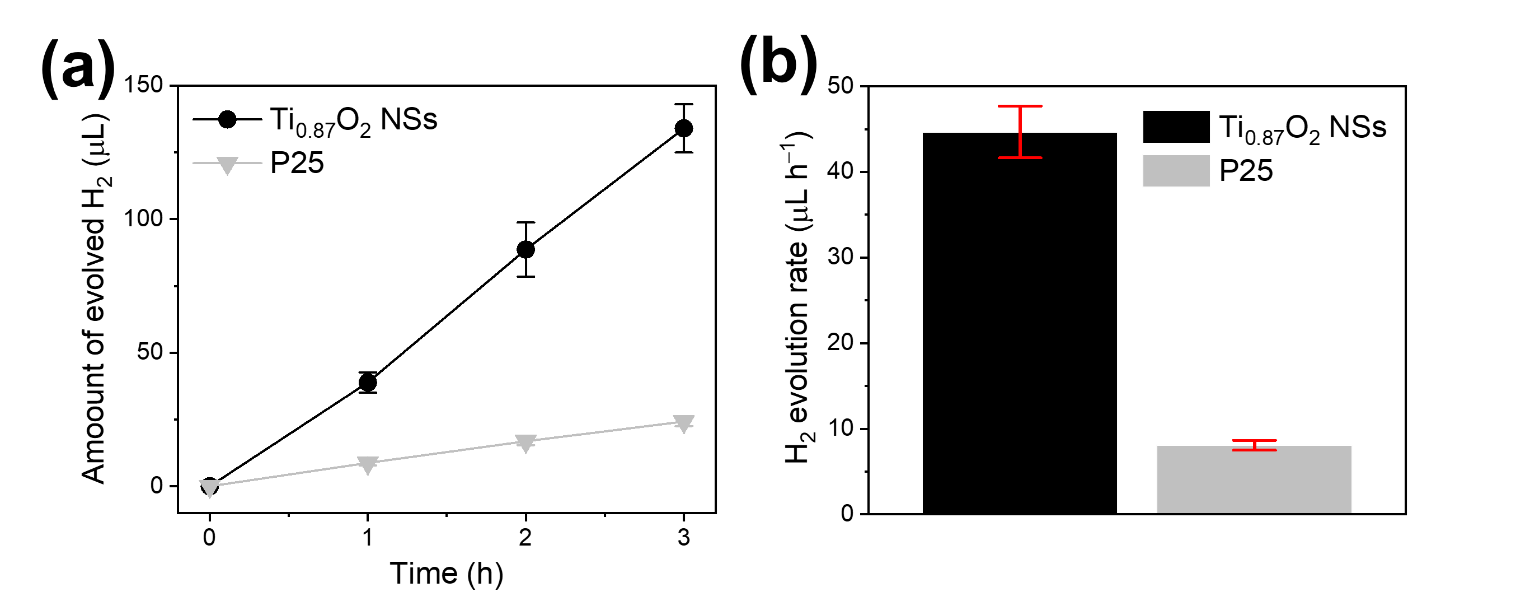
**

**Fig. S6.** Photocatalytic hydrogen evolution performance for Ti_0.87_O_2_ NSs and P25 under 275 nm LED 3 h illumination.

**
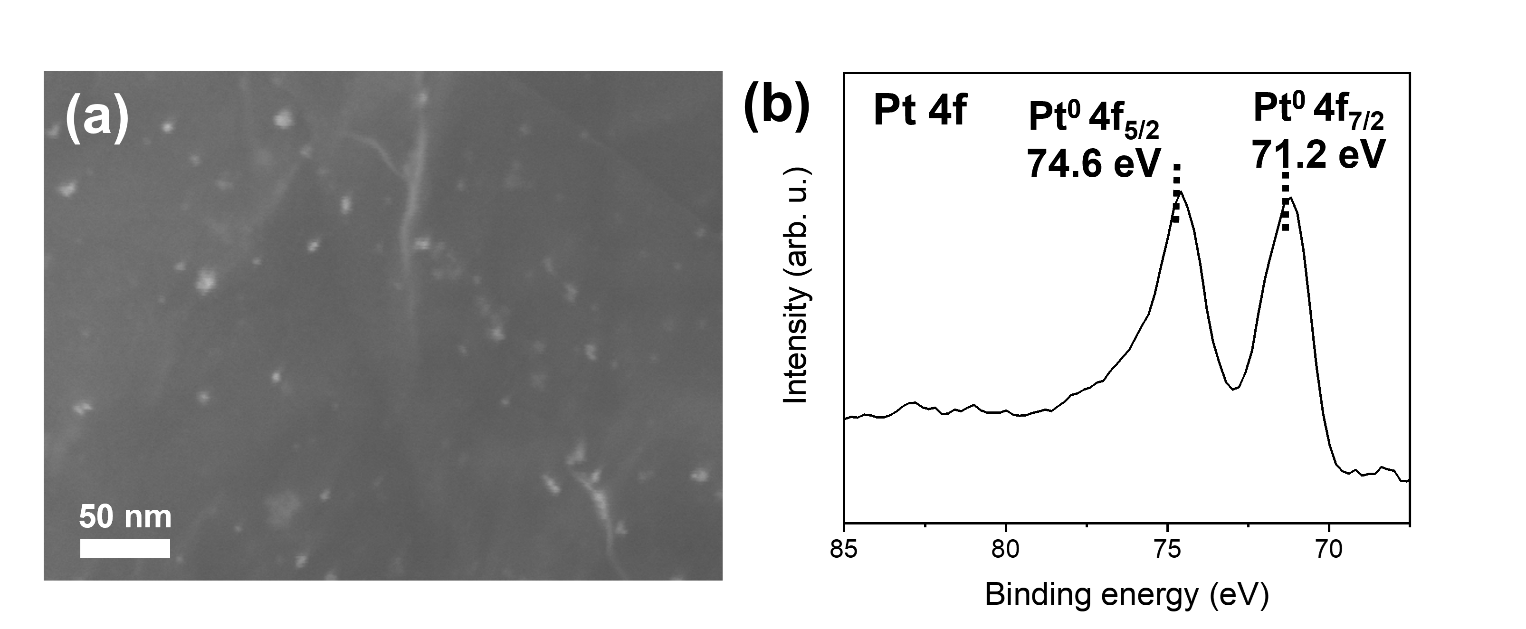
**

**Fig. S7.** (a) SEM image and (B) Pt 4f XPS spectrum of Pt NPs/Ti_0.87_O_2_ NSs.

**
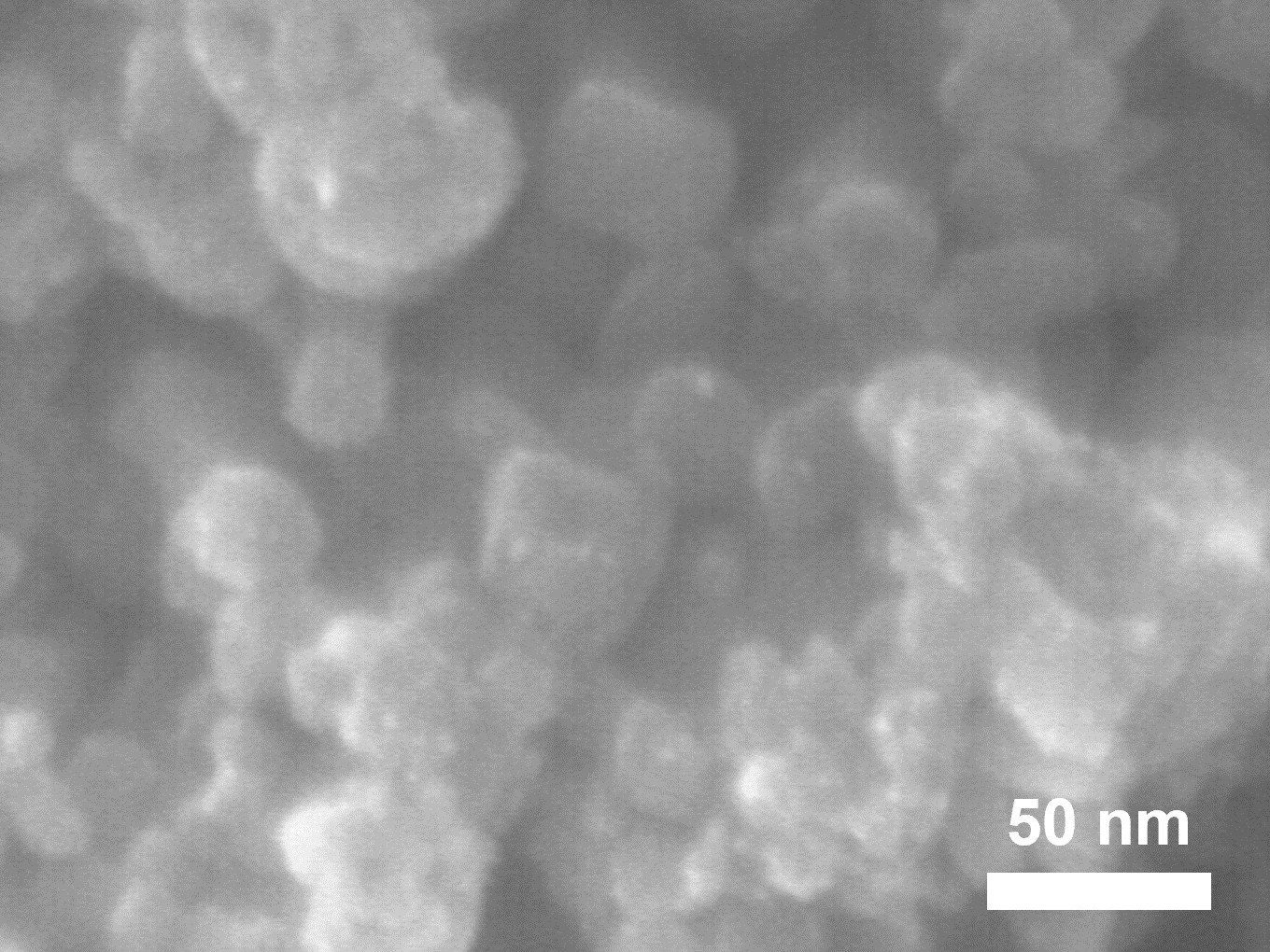
**

**Fig. S8.** SEM image of Pt SAs/P25 after photocatalysis.

**
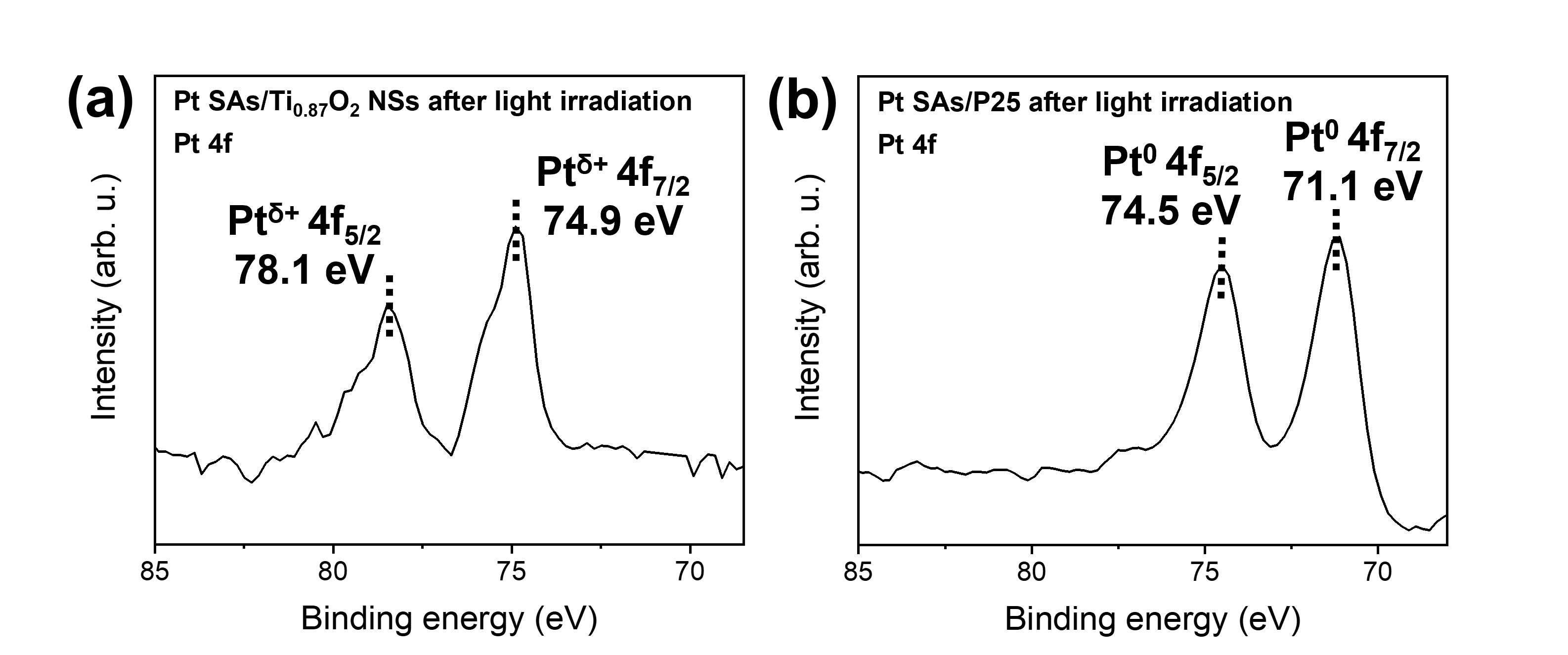
**

**Fig. S9.** Pt 4f XPS spectra of Pt SAs/Ti_0.87_O_2_ NSs and Pt SAs/P25 after 275 nm LED 5 min irradiation.


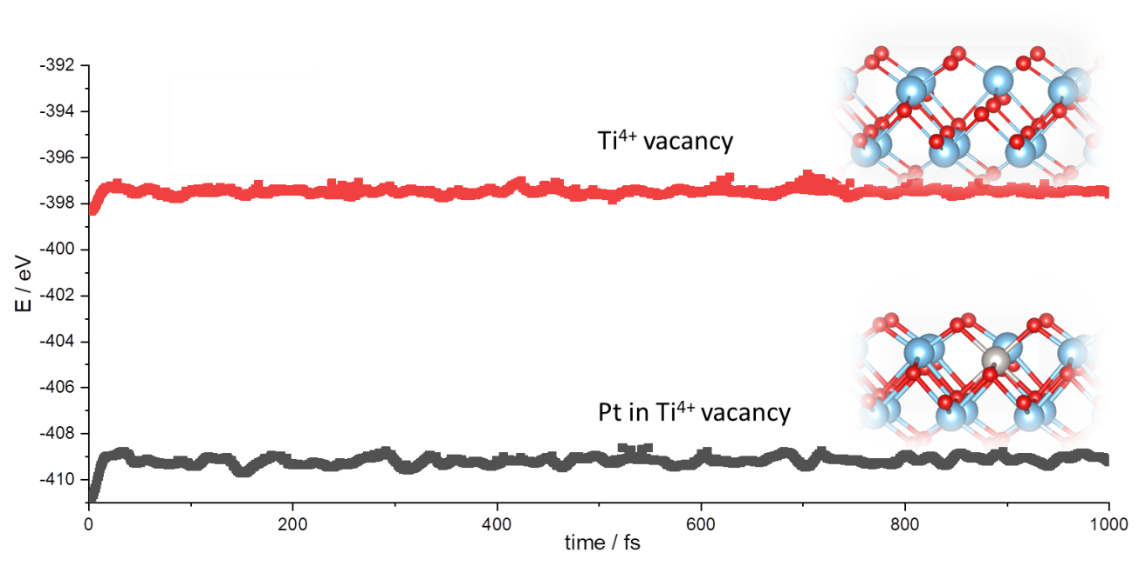


**Fig. S10.** Total energy of the Ti-deficient TiO_2_ NS and Ti-deficient TiO_2_ NS with Pt SA embedded in the Ti^4+^ vacancy, during molecular dynamics simulation at 298 K.


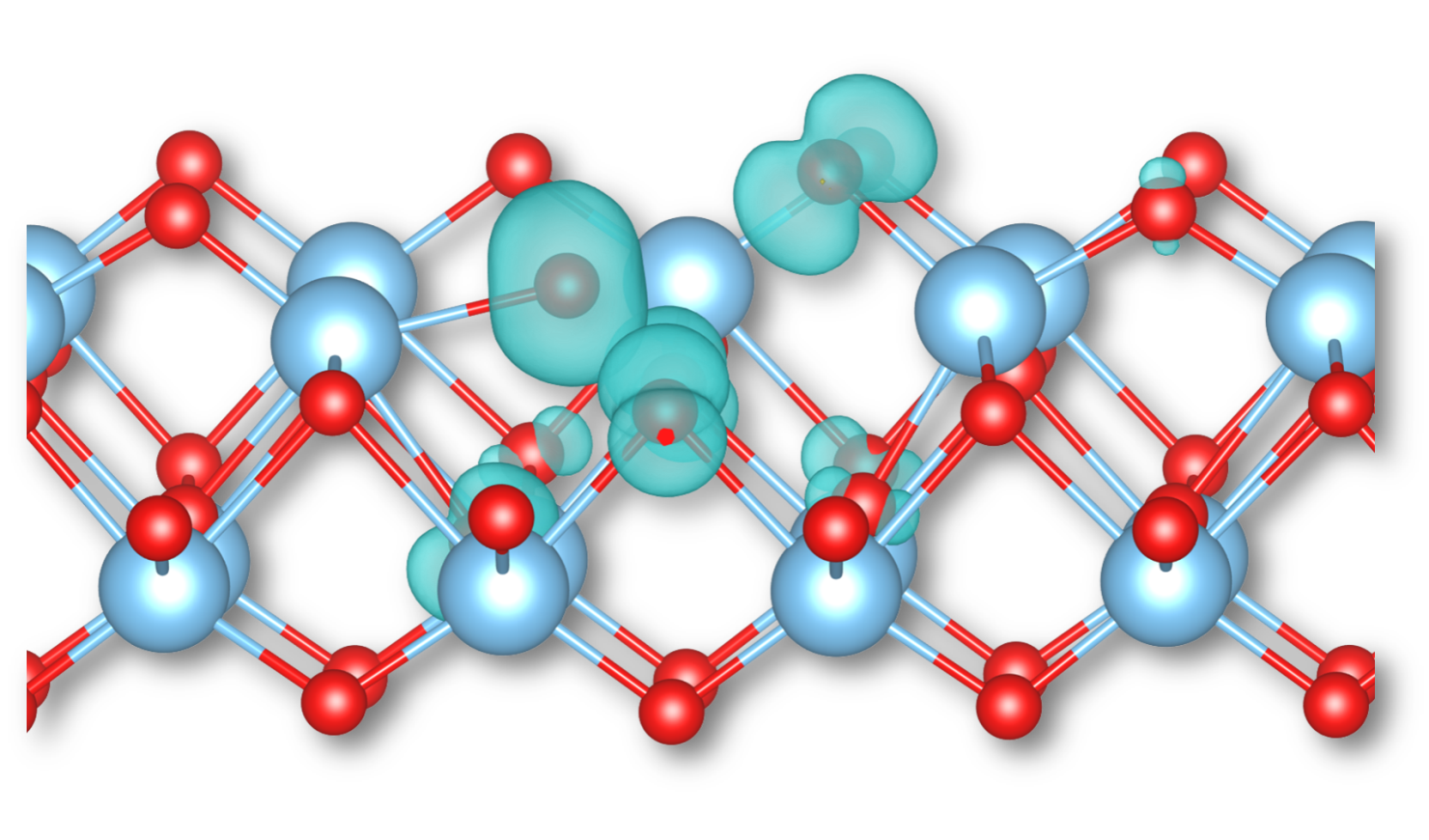


**Fig. S11.** Spin density around the Ti^4+^ vacancy in Ti-deficient TiO_2_ NS (isosurface value 0.015 e A^−3^) extracted from molecular dynamics simulations at 298 K.
